# Association of dietary fiber intake with newly-diagnosed type 2 diabetes mellitus in middle-aged Chinese population

**DOI:** 10.1186/s12937-021-00740-2

**Published:** 2021-09-27

**Authors:** Fubi Jin, Jinghong Zhang, Long Shu, Wei Han

**Affiliations:** 1 Department of Endocrinology, Zhejiang Hospital, Lingyin Road Number 12, Xihu District, Hangzhou, 310013 Zhejiang People’s Republic of China; 2 Department of Nutrition, Zhejiang Hospital, Lingyin Road Number 12, Xihu District, Hangzhou, 310013 Zhejiang People’s Republic of China; 3 Department of Cardiovascular Medicine, Zhejiang Hospital, Lingyin Road Number 12, Xihu District, Hangzhou, 310013 Zhejiang People’s Republic of China

**Keywords:** Dietary fiber, Glycemic control, Type 2 diabetes mellitus, Newly-diagnosed, Middle-aged and elderly Chinese

## Abstract

**Background:**

Epidemiological evidence concerning dietary fiber on newly-diagnosed type 2 diabetes mellitus (T2DM) is sparse. Therefore, the purpose of this study was to investigate the relationship between dietary fiber intake and newly-diagnosed T2DM in a middle-aged Chinese population.

**Methods:**

Using data from the Hangzhou Nutrition and Health Survey collected between June 2015 and December 2016, we investigated the associations between dietary patterns and the risk of chronic non- communicable diseases. Anthropometric measurements and samples collection for biochemical assays are conducted by the well-trained staff and nurse, respectively. Multivariable logistic regression analysis was used to analyze the effect of dietary fiber intake on the risk of newly-diagnosed T2DM in crude and adjusted models.

**Results:**

Among 3250 participants, 182 (5.6%) people were identified as newly-diagnosed T2DM. Pearson correlation coefficients revealed a significant inverse association of total dietary fiber with BMI, SBP, DBP, HbA1c and LDL-C in all participants, participants with and without T2DM (*P* < 0.05). Compared with the study participants in the first quartile (Q1, the lowest consumption)of dietary fiber intake, participants in the fourth quartile (Q4) had a lower prevalence of newly-diagnosed T2DM(OR = 0.70; 95%CI:0.49-1.00; *P* < 0.05), after adjustment for potential confounders.

**Conclusions:**

In this middle-aged Chinese population, higher intake of dietary fiber was significantly associated with lower risk of newly-diagnosed T2DM. However, our findings need to be confirmed in future large-scale prospective studies.

## Introduction

Type 2 diabetes mellitus(T2DM) is a major non-communicable chronic disease that affects the health, economy and well-being of populations worldwide [1]. Based on the statistics reported by the International Diabetes Federation(IDF), 449 million people had T2DM around the world, and this number is estimated to reach 702 million by 2045 [2]. Currently, Asia has emerged as the major area with a rapidly developing T2DM. In China, a low- and middle-income country, T2DM has been recognized as a major health problem and the prevalence has rapidly increased since 1980 [3]. Zimmet and his colleagues reported that an estimated> 120 million Chinese people had T2DM in 2017 [4]. It is well- known that T2DM is a multifactorial chronic disease associated with genetic factors, unhealthy lifestyles (e.g. physical inactivity and smoking) and dietary factors [5, 6].

During the past several decades, dietary factors have been shown to play an important role in prevention and management of T2DM [6]. Among these dietary factors, dietary fiber has attracted much attention of researchers in glycemic control in T2DM patients [7]. So far, a number of epidemiological studies have documented a link between dietary fiber consumption and the risk of T2DM [8–13], but these findings are controversial. Whilst some studies have shown the protective association between higher dietary fiber intake and the risk of T2DM [8, 10, 12, 13], others also showed no significant associations [9, 11]. Furthermore, to the authors’ knowledge, no previous epidemiological study has been conducted to investigate dietary fiber intake in relation to newly-diagnosed T2DM risk in the Chinese population. In view of the current paucity of data in this area, we took advantage of a large population-based cross-sectional study performed in Eastern China, to determine the association between dietary fiber intake and the risk of newly-diagnosed T2DM in a middle-aged population.

## Subjects and methods

### Study population

**Fig. 1:**
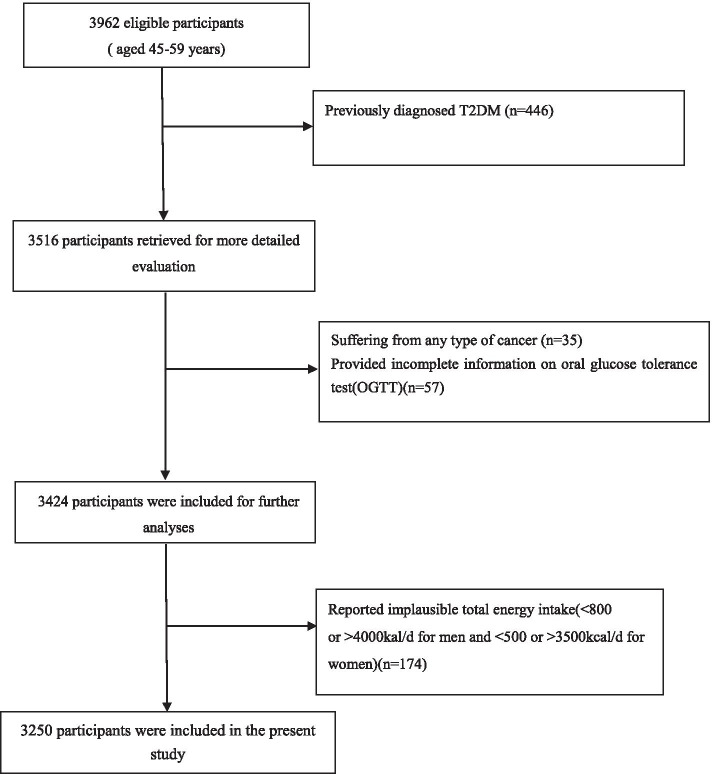
Flow chart of article screening and selection process

This cross-sectional study was conducted in Hangzhou city, the capital of Zhejiang Province, East China, from June 2015 to December 2016. Details on participant recruitment have been published elsewhere [14]. Briefly, we used a multistage cluster random-sampling method to select a sample in the middle-aged Chinese population. A total of 3962 eligible participants were invited to attend a health examination at the Medical Center for Physical Examination, Zhejiang Hospital, where the participants were face-to-face interviewed by trained interviewers using written questionnaires. For our analyses, the exclusion criteria were previously diagnosed T2DM (*n* = 446), incomplete information on oral glucose tolerance test(OGTT)(*n* = 57), suffering from any type of cancer (*n* = 35) and implausible total energy intake(< 800 or > 4000kal/d for men and < 500 or > 3500 kcal/d for women)(*n* = 174). After these exclusions, 3250 participants(1674 women and 1576 men) were included in the final analysis. A flow chart detailing the process of study selection is shown in Fig. 1. Written informed consent was obtained from all study participants before data collection.

### Assessment of dietary intake

Dietary intake information was obtained through a validated semi-quantitative food frequency questionnaire(FFQ). The FFQ specifies 138 food items, covering 90% of food items commonly consumed in Zhejiang Province [14]. Briefly, for each food item, participants were requested to recall their usual frequency of consumption in previous 12 months and the estimated portion size by making comparisons with the specified reference portion. Food pictures with portion sizes were provided to aid participants to evaluate their food intake by well-trained interviewers. For liquid foods such as milk, juice and beverage, the amount of intake was reported in milliliter (ml) and was transformed into gram in the analysis. The frequency of consumption for each food item had nine responses that ranged from “never or almost never” to “3 times/day”. Then, each food item was converted into an average daily consumption. Nutrient content from the Chinese Food Composition Tables was applied to estimate nutrient intake from all food items and groups, and to obtain total dietary fiber for most food items [15].

### Assessment of blood pressure

Before measuring blood pressure, participants were asked to rest for 5-10 min in the sitting position. Then, blood pressure was measured two times by a trained nurse using an automatic monitor (Model: OMRON HEM-780), and the mean values of two readings were documented and used in the analysis.

### Assessment of biomarker

All blood samples were collected after overnight fasting for 12 h, and allowed to clot at room temperature for 1-2 h. A 1 ~ 1.5 ml venous blood specimen was collected in a vacuum tube containing sodium fluoride for the measurement of plasma glucose, lipids and HbA1c. Then, plasma was separated by centrifugation for 15 min at 3000r.p.m., and stored at 4 °C until assayed. High-performance liquid chromatography was used to measure HbA1c (HPLC, BioRad variant). Total plasma cholesterol and triglyceride were measured using standard enzymatic methods; HDL-cholesterol was separated using dextran sulphate, and LDL-cholesterol was calculated using the Friedewald formula. These biochemical assays are conducted in the Medical Center for Physical Examination, Zhejiang Hospital, using an Automatic Biochemical Analyzer (Hitachi 7180, Hitachi, Ltd., Tokyo, Japan).

### Assessment of anthropometric measurements

Anthropometric measurements were obtained and included weight, height, and waist circumference (WC). Weight was measured using a balanced digital scale to the nearest 0.1 kg (without shoes and in light clothes). Height was measured to the nearest 0.1 cm with participants in standing position and without shoes. Body mass index(BMI) was calculated as weight in kilograms(kg) divided by the square of height in meters(kg/m^2^). WC was measured half-way between the last rib and the iliac crest, and hip circumstance was measured at the maximum level over light clothing, by using an anthropometric tape [16]. All these measurements were conducted at the interview according to the standard protocol.

### Assessment of other variables

Data on general information including smoking status, educational level, and income were collected using a self-administered questionnaire. Smoking status was categorized into never smokers, current smokers, and former smokers; Educational level was categorized into middle school or below, high school, college or above. In addition, physical activity level was assessed by trained interviewers using the International Physical Activity Questionnaire (IPAQ) [17], and expressed as metabolic equivalent hours per week (MET-h/week). The IPAQ included work-related activity, housework activity, leisure, transport-related activity, exercise, sitting posture, and sleeping time. Finally, total energy intake was estimated through the semi-quantitative FFQ, expressed in kilocalorie per day (kcal/day).

### Definition of T2DM

T2DM was defined as the presence of any one of the following: (1)FPG ≥ 7.0 mmol/L on at least two separate occasions, or an oral glucose tolerance test(OGTT) with a value≥11.1 mmol/L; (2) current use of insulin or oral hypoglycemic agents; or (3) a positive response to the question: have you ever been diagnosed with diabetes by a doctor? [6].

### Statistical analysis

Data were analyzed across quartiles of dietary fiber intake, and the results for continuous variables are presented as mean ± standard deviation(SD) or median and interquartile range(IQR), and categorical variables are presented as sum and percentages. Variables were examined for normality (normal plots), and non-normally distributed variables were log transformed. The Independent-Samples t test (for normal distributed variables) or the Mann-Whitney test (if non- parametric tests were required) was used evaluate the significant differences in continuous variables, while the chi-squared test was used to evaluate the significant differences in categorical variables. Correlations of dietary fiber with HbA1c, BP, BMI, WC and other variables were examined using Pearson correlation coefficients. Multivariable logistic regression models were used to evaluate the association between dietary fiber and risk of newly-diagnosed T2DM, after correction for potential confounders. Model 1 was adjusted for gender (male/female) and age (continuous); Model 2 was further adjusted for education level(<high school, high school, >high school), physical activity level (light, moderate, heavy), smoking status(never, current, former), family history of diabetes and hypertension, alcohol intake and total energy intake (continuous); Model 3 was additionally adjusted for BMI. All statistical analyses were performed using IBM SPSS Statistics, version 24.0(IBM Corp., Armonk, NY, USA). All *P*-values are two-tailed and statistical significance was set at the conventional cutoff of *P* < 0.05.

## Results

Of the 3250 participants, 48.5%(1576) were men. The mean age was 47.2 ± 9.0y (range: 45-59 y) for T2DM and 52.1 ± 8.7y (range: 47-59y) for non-T2DM. Overall prevalence of newly-diagnosed T2DM in our study population was 5.6%. Baseline and demographic characteristics of the study participants by newly-diagnosed T2DM status are shown in Table 1. There were significant differences between participants with and without T2DM by age, BMI, WC, fiber intake(fruit and vegetables fiber; total fiber), HbA1c, total energy intake, smoking status, education and the prevalence of obese (*P* < 0.05).

**Table 1:** Demographic characteristics of the study participants by newly-diagnosed T2DM status

Variables	Participants with T2DM(*n* = 182)	Participants without T2DM (*n* = 3068)	Significance^*^
Demographic			
Age (years)	47.2 ± 9.0	52.1 ± 8.7	*P* < 0.0001
BMI(kg/m^2^)	25.1 ± 5.7	23.7 ± 4.1	*P* < 0.0001
WC(cm)	89.5 ± 12.4	83.0 ± 11.0	*P* < 0.0001
Cereal fiber(g)	6.8(3.7-10.2)	7.1(4.2-9.8)	0.58
Fruit and vegetable fiber(g)	9.0(7.2-16.4)	10.6(8.5-19.7)	< 0.05
Total fiber(g)	15.2(11.0-20.5)	17.5(14.2-28.3)	< 0.01
HbA1c (%)	7.9 ± 1.2	5.4 ± 0.5	< 0.0001
Total energy intake(kcal/d)	2092.5 ± 603.7	1758.0 ± 528.4	< 0.0001
Gender			χ^2^ = 3.785
Men	101(55.4)	1475(48.1)	*P* = 0.052
Women	81(44.6)	1593(51.9)	
Smoking status (%)			χ^2^ = 11.257
Never	72(39.6)	1604(52.3)	*P* < 0.01
Former	74(40.6)	963(31.4)	
current	36(19.8)	501(16.3)	
Education (%)			χ^2^ = 8.388
< High school	34(18.7)	745(24.3)	*P* < 0.05
High school	98(53.8)	1733(56.5)	
> High school	50(27.5)	590(19.2)	
Income per month			χ^2^ = 1.735
≤ 2000 (RMB)	76(41.7)	1144(37.3)	*P* = 0.420
2000-5000 (RMB)	86(47.3)	1519(49.5)	
> 5000 (RMB)	20(11.0)	405(13.2)	
Obesity (%)			χ^2^ = 7.464
Yes	30(16.5)	310(10.1)	*P* < 0.001
No	152(83.5)	2758(89.9)	
Hypertension(%)			χ^2^ = 0.193
Yes	37(20.3)	666(21.7)	*P* = 0.661
No	145(79.7)	2402(78.3)	
Physical activity(%)			χ^2^ = 4.534 *P* = 0.104
Light	128(70.3)	2062(67.2)	
Moderate	45(24.7)	709(23.1)	
Heavy	9(5.0)	297(9.7)	

Categorical variables are presented as sum and percentages, and continuous variables are presented as Mean ± SD and median (IQ range). Abbreviation: *T2DM* Type 2 diabetes mellitus, *BMI* Body mass index, *WC* Waist circumference, *HbA1c* Hemoglobin A1c, *RMB* Ren min bi. *P values for continuous variables (for the normal distributed variables, Independent-Samples t test was used to assess the significant differences; If not, the Mann-Whitney test was required) and for Categorical variables (chi-square test)

General characteristics of 3250 participants of the Nutrition and Health Survey according to dietary fiber intake categories are shown in Table 2. Compared with those in the lowest quartile(Q1), participants in the highest quartile of dietary fiber intake(Q4) were more likely to be older, women, never-smoker, and to have lower BMI, educational level, income and total energy intake, and higher physical activity level and lower prevalence of obesity, hypertension and T2DM(*P* < 0.05).

**Table 2:** General characteristics of 3250 participants of the Nutrition and Health Survey according to dietary fiber intake categories

Characteristics	Dietary fiber intake				*P*-value^*^
	Q1(2.1 ~ 9.8 g) (*n* = 812)	Q2(~ 13.5 g) (*n* = 813)	Q3(~ 21.4 g) (*n* = 813)	Q4(~ 55.7 g) (*n* = 812)	
Age(years)	48.5 ± 1.7	51.3 ± 1.4	50.9 ± 1.5	52.4 ± 1.9	< 0.0001
BMI(kg/m^2^)	26.2 ± 4.5	25.1 ± 4.7	24.9 ± 4.1	23.6 ± 5.3	< 0.0001
Obesity[n(%)]	138(17.0)	89(10.9)	67(8.2)	46(5.7)	< 0.0001
Hypertension[n(%)]	203(22.8)	174(20.8)	176(21.9)	150(20.7)	0.017
T2DM[n(%)]	59(7.3)	50(6.2)	41(5.0)	32(3.9)	< 0.0001
Gender (%)					χ2 = 7.331 0.062
Men	416(51.2)	409(50.3)	369(45.4)	382(47.0)	
Women	396(49.8)	404(49.7)	444(54.6)	430(53.0)	
Smoking status(%)					χ2 = 60.388 < 0.0001
Never-smokers	379(46.7)	403(50.0)	398(49.0)	496(61.1)	
Former-smokers	273(33.6)	262(32.2)	258(31.7)	244(30.0)	
Current-smoker	160(19.7)	148(17.8)	157(19.3)	72(8.9)	
Education[n(%)]					χ2 = 52.182 < 0.0001
< High school	137(16.9)	217(26.7)	200(24.6)	225(27.7)	
High school	458(56.4)	455(56.0)	468(57.6)	450(55.4)	
> High school	217(26.7)	141(17.3)	145(17.8)	137(16.9)	
Income/per month/ person (%)					χ2 = 29.061 < 0.0001
< 2000(RMB)	275(33.9)	297(36.5)	307(37.8)	341(42.0)	
2000-5000(RMB)	412(50.7)	385(47.4)	410(50.4)	398(49.0)	
> 5000(RMB)	125(15.4)	131(16.1)	96(11.8)	73(9.0)	
Physical activity (METs-h/week)	23.2 ± 2.88	22.9 ± 2.17	26.5 ± 2.91	30.6 ± 3.09	< 0.0001
Total energy intake (kcal/d)	2194.5 ± 524.7	2203.2 ± 549.6	2094.8 ± 519.7	1807.8 ± 512.0	< 0.0001

Categorical variables are presented as sum and percentages, and continuous variables are presented as Mean ± SD. Abbreviation: *Q1* Quartile 1, *BMI* Body mass index, *T2DM* Type 2 diabetes mellitus, *RMB* Ren min bi. * *P* values were derived by chi-square tests for categorical variables and ANOVA tests for continuous variables

Pearson bivariate correlation of total dietary fiber with other variables was shown in Table 3. Total dietary fiber exhibited an inverse correlation with BMI, SBP, DBP, HbA1c and LDL-C in all participants, participants with and without T2DM(*P* < 0.05). WC, WHR, and TG was inversely associated with total dietary fiber in both all participants and participants without T2DM(*P* < 0.05). TC was inversely associated with total dietary fiber in both all participants and participants with T2DM(*P* < 0.01). FG was inversely associated with total dietary fiber in participants without T2DM(*P* < 0.05). Besides, age was positively associated with total dietary fiber in all participants as well as participants without T2DM(*P* < 0.01).

**Table 3:** Pearson bivariate correlation coefficients of total dietary fiber with other variables

Variables	All participants(*n* = 3250)		Participants with T2DM (*n* = 182)		Participants without T2DM (*n* = 3068)	
	r	*P*-value	r	*P*-value	r	*P*-value
Age (y)	0.128	0.000	−0.087	0.054	0.186	0.000
BMI(kg/m^2^)	− 0.151	0.000	0.209	0.000	−0.170	0.000
WC(cm)	−0.087	0.050	−0.002	0.056	−0.099	0.032
WHR	−0.072	0.046	0.003	0.078	−0.100	0.049
SBP(mm Hg)	−0.105	0.001	−0.092	0.045	−0.122	0.000
DBP(mm Hg)	−0.149	0.000	−0.089	0.037	−0.125	0.001
HbA1c	−0.091	0.01	−0.105	0.006	−0.112	0.000
FG(mmol/L)	−0.042	0.336	−0.039	0.425	−0.074	0.031
TG(mmol/L)	−0.067	0.044	0.006	0.890	−0.106	0.007
TC(mmol/L)	−0.104	0.009	−0.166	0.000	−0.077	0.060
HDL-C(mmol/L)	0.054	0.126	0.001	0.094	0.087	0.066
LDL-C(mmol/L)	−0.144	0.009	−0.113	0.038	−0.101	0.049

*BMI* Body mass index, *WC* Waist circumference, *WHR* Waist-to-hip ratio, *SBP* Systolic blood pressure, *DBP* Diastolic blood pressure, *HbA1c* Hemoglobin A1c, *FG* Fasting glucose, *TG* Triglycerides, *TC* Total cholesterol, *HDL-C* High density lipoprotein-cholesterol, *LDL-C* Low density lipoprotein-cholesterol

The association between dietary fiber intake and newly-diagnosed T2DM risk by logistic regression analysis is presented in Table 4. After correction of confounders, participants in the highest quartile of dietary fiber had lower odds of the T2DM(OR = 0.70; 95%CI:0.49-1.00; *P* < 0.05) than did those in the lowest quartile.

**Table 4:** Multivariable adjusted odds ratio(95%CI) for newly-diagnosed T2DM according to dietary fiber intake

	Dietary fiber intake			
	Q1(*n* = 812)	Q2(*n* = 813)	Q3(*n* = 813)	Q4(*n* = 812)
Incident cases of T2DM	69	33	37	43
Model 1	1(ref.)	0.76 (0.52-1.08)	0.72 (0.48-0.96)*	0.51 (0.34-0.75)**
Model 2	1(ref.)	0.89 (0.67-1.06)	0.88 (0.64-1.03)	0.59 (0.39-0.82)*
Model 3	1(ref.)	0.96 (0.81-1.49)	0.93 (0.71-1.22)	0.70(0.49-1.00)*

Abbreviation: *T2DM* type 2 diabetes mellitu; Dietary fiber quartiles: Quartile 1[mean: 7.6 g(range:2.1-9.8)]; Quartile 2[mean: 11.5 g(range:9.8-13.5)]; Quartile 3[mean: 15.9 g(range:13.5-21.4)]; Quartile 4[mean: 26.3 g(range:21.4-55.7)]; *P < 0.05, **P < 0.01; Model 1: adjusted for age (continuous) and gender. Model 2: additionally adjusted for education level(<high school, high school, >high school), physical activity (continuous), smoking status(never, current, former), family history of diabetes and hypertension, alcohol intake and total energy intake (continuous);Model 3: additionally adjusted for BMI

## Discussion

Epidemiological evidence on the association of dietary fiber consumption and newly-diagnosed T2DM risk in Chinese population is scant. To the best of our knowledge, this is the first study investigating the association of dietary fiber intake with the risk of newly-diagnosed T2DM in this middle-aged Chinese population. Our results indicated that higher dietary fiber intake was significantly associated with reduced risk of newly-diagnosed T2DM. Over the last couple of decades, China has experienced a significant nutrition transition that more and more Chinese adults predominantly choose Western diet, with the increased consumption of red and processed meat, and decreased consumption of dietary fiber [18]. In fact, dietary fiber has been implicated as a nutrient of public health concern worldwide [19].Currently, an increased intake of dietary fiber has also been recommended by some international organizations [20, 21]. The American Diabetes Association recommends that dietary fiber intake in patients with diabetes should match the recommendations for the general population, to increase the total fiber intake to 14 g per 1000 cal, or 25 g/d for women and 38 g/d for men [21]. Besides, the results from the Shanghai Women’s study showed that the highest quintile of dietary fiber intake was 16.3 g/d [22]. In our study population, we observed an average intake of dietary fiber of 11.7 g/d, which is less than half the recommendations of 25 g/d for Chinese adults [7].

In our analyses, we observed an inverse association of dietary fiber intake with risk of newly- diagnosed T2DM in highest versus lowest categories, after adjustment for the potential confounding variables. Our findings are similar to the findings from a previous meta-analysis [7], which indicated that higher intake of dietary fiber was inversely associated with the risk of T2DM. Also, in a meta-analysis of randomized clinical trials reported by Silva et al., higher fiber intake (> 42.5 g/d) was found to significantly decrease the levels of HbA1c and FPG in adults with T2DM [23]. Similarly, Jiang and colleagues found that increasing fiber intake may be an effective approach to reduce HbA1c level, improving glycemic control among in Chinese diabetic patients [24]. In another systematic review, foods rich in soluble fiber, e.g. beta-glucans, were shown to improve glycemia in diabetes patients [25]. Thus, several mechanisms have been proposed to explain the favorable effects of dietary fiber intake on the risk of newly-diagnosed T2DM. First, high intake of dietary fiber could promote the feeling of fullness and reduce the intake of energy-dense foods, resulting in a decreased risk of overweight/ obesity, which is an estimated risk factor for T2DM [26]. Second, consumption of dietary fiber, especially soluble fiber could delay gastric emptying and decrease absorption of macronutrients, resulting in lower postprandial blood glucose and insulin level [27]. Third, evidence supported that higher intake of dietary fiber was inversely associated with a reduction in inflammatory markers, e.g. interleukin-6 and tumor necrosis factor α(TNF-α) that are central in the initiation and progression of T2DM [9]. Fourth, dietary fiber intake has been found to be inversely associated with insulin resistance, which has been reported to be the risk factor for T2DM [28]. Liese et al., has shown that dietary fiber was associated with insulin sensitivity, and improved the ability to delay the absorption of carbohydrates and secrete insulin adequately to overcome insulin resistance, resulting in lower postprandial blood glucose and insulin levels [29]. Finally, in a new randomized controlled trial, Zhao and his colleagues showed that in patients with T2DM a high-fiber diet promotes SCFA-producing gut bacteria, which in turn normalizes levels of HbA1c via an increase in the production of glucagon-like peptide 1 (GLP1) [30].Overall, the importance of increasing intake of dietary fiber must be emphasized in the T2DM population (particularly in newly-diagnosed T2DM).

### Strengths and limitations

There are several strengths and limitations in our study. First, to our knowledge, this is the first study in a large sample of adults in Eastern China to reveal the association of dietary fiber intake with the risk of newly-diagnosed T2DM. Our study provided further evidence for understanding the relationship between dietary fiber intake and newly-diagnosed T2DM risk, and contributed to reducing the incidence of T2DM through increasing dietary fiber intake. Second, dietary fiber intake was collected using a validated semi-quantitative FFQ for the Chinese population. The reliability and validity of this FFQ has been reported in our previous study [14]. Third, we also have adjusted for some known and suspected potential confounders in our analyses. Nonetheless, several limitations of this study should be addressed. First, due to the nature of the cross-sectional design, we could not elucidate the cause and effect relationship between dietary fiber intake and the risk of newly-diagnosed T2DM. Future studies with longitudinal designs are needed to confirm these findings. Second, dietary fiber intake was estimated by the use of an FFQ at a single time point, which inevitably led to a degree of misclassification. Besides, we also didn’t calculated detailed nutrients and energy intakes from dietary habits. Third, although our statistical analyses were adjusted for several potential confounding variables, the possibility of residual confounding or unmeasured confounders cannot be completely ruled out. Finally, our study sample represents a highly selected part of Chinese population (the middle-aged population in Zhejiang Province, East China), limiting the generalizability of our results.

## Conclusions

In conclusion, we found that higher intake of dietary fiber was associated with a decreased risk of newly-diagnosed T2DM in a middle-aged Chinese population. These findings support current dietary recommendations to increase intake of dietary fiber as a primary preventive measure against T2DM. Meanwhile, our findings also add to the evidence that increasing fiber intake may be an effective approach to improve glycemic control among newly-diagnosed T2DM patients. However, future prospective studies are required to confirm these associations.

## Data Availability

Not applicable.

## Abbreviations

T2DM: Type 2 diabetes mellitus
OR: Odds ratio
CI: Confidence interval
FFQ: Food frequency questionnaire
BMI: Body mass index
WC: Waist circumference
WHR: Waist-to-hip ratio
SBP: Systolic blood pressure
DBP: Diastolic blood pressure
HbA1c: Hemoglobin A1c
FG: Fasting glucose
TG: Triglycerides
TC: Total cholesterol
HDL-C: High density lipoprotein-cholesterol
LDL-C: Low density lipoprotein-cholesterol
RMB: Ren min bi
IPAQ: International Physical Activity Questionnaire
OGTT: Oral glucose tolerance test
